# A Lightweight Authentication and Key Agreement Protocol for IoT-Enabled Smart Grid System

**DOI:** 10.3390/s23083991

**Published:** 2023-04-14

**Authors:** Chen Chen, Hua Guo, Yapeng Wu, Bowen Shen, Mingyang Ding, Jianwei Liu

**Affiliations:** 1 School of Cyber Science and Technology, Beihang University, Beijing 100191, China; 2 State Key Laboratory of Cryptology, Beijing 100878, China; 3 State Key Laboratory of Software Development Environment, Beihang University, Beijing 100191, China; 4 Sino-French Engineer School, Beihang University, Beijing 100191, China

**Keywords:** smart grid, IoT, lightweight, authentication and key agreement protocol, insider attack

## Abstract

The IoT-enabled Smart Grid uses IoT smart devices to collect the private electricity data of consumers and send it to service providers over the public network, which leads to some new security problems. To ensure the communication security in a smart grid, many researches are focusing on using authentication and key agreement protocols to protect against cyber attacks. Unfortunately, most of them are vulnerable to various attacks. In this paper, we analyze the security of an existent protocol by introducing an insider attacker, and show that their scheme cannot guarantee the claimed security requirements under their adversary model. Then, we present an improved lightweight authentication and key agreement protocol, which aims to enhance the security of IoT-enabled smart grid systems. Furthermore, we proved the security of the scheme under the real-or-random oracle model. The result shown that the improved scheme is secure in the presence of both internal attackers and external attackers. Compared with the original protocol, the new protocol is more secure, while keeping the same computation efficiency. Both of them are 0.0552 ms. The communication of the new protocol is 236 bytes, which is acceptable in smart grids. In other words, with similar communication and computation cost, we proposed a more secure protocol for smart grids.

## 1. Introduction

With the rapid growth of Internet of Things (IoT), the IoT-enabled smart grid is gradually replacing the traditional power grid and becoming one of the important infrastructures in real society [1,2,3,4]. The IoT-enabled smart grid integrates wireless sensor networks into the power system, and obtains physical information such as grid operation status and parameters through wireless sensor networks. In smart grids, the wireless sensor network has become a useful supplement to the production, transmission, distribution and consumption of electric energy.

**Figure 1:**
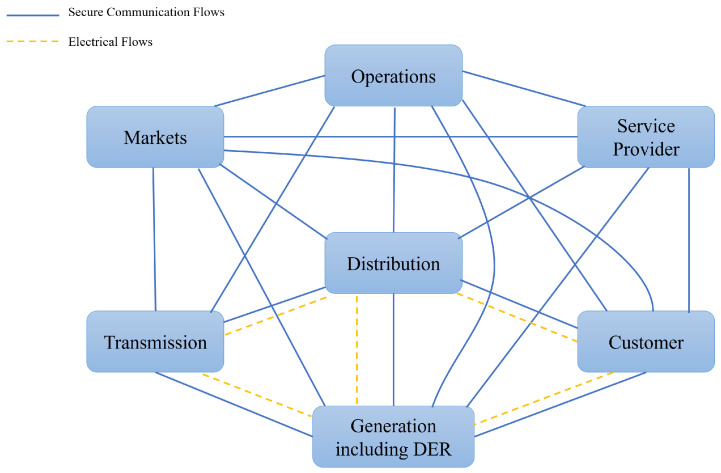
NIST smart grid conceptual network model.

According to the latest NIST Framework and Roadmap for Smart Grid Interoperability Standards 4.0 [5], released in 2021, the smart grid model can be roughly abstracted into seven domains: customer, markets, service provider (SP), operations, generation (including DER), transmission and distribution, as shown in Figure 1. The solid blue line indicates secure communication flows and the dotted yellow line indicates electrical flows. The Customer is the end user of electricity, who may also generate, store, and manage the use of energy, which relies on smart meters (SMs) to access the smart grid. The Markets are the facilitators and participants in electricity markets and other economic mechanisms used to drive action and optimize system outcomes.The Service Provider is the organization providing services to electrical customers and to utilities.The Operations are the managers of the movement of electricity. The Generation Including DER is the producer of electricity, and may also store energy for later distribution. Transmission refers to the carriers of high voltage electricity over long distances. Distribution is the distributor of electricity to and from customers.

The smart grid (SG) is superior to the traditional grid in several aspects [6], such as flexible control, efficient operation, convenient use, etc.. However, as mentioned above, the SM is deployed on the side of consumers, so it is easy for most people to contact the SM, which gives a great opportunity to malicious attackers [7,8]. On the contrary, due to the large number of SMs, it may be difficult for service providers to carry out effective security monitoring and physical protection. In other words, the security of the smart grid relies on the security of the communication protocol used in the communication between SMs and SPs in the power grid, and whether the identity authentication and information transmission can be carried out securely. On 23 December 2015, some attackers successfully forged identity authentication and attacked the control system of the smart grid by taking advantage of the loopholes in the smart grid of Ukraine’s energy supply company, which resulted in unexpected hours of large-scale blackout and great damage to the normal management of the society [9].

### 1.1. Related Work

Many researchers have paid attention to the authentication and key agreement protocols under smart grid circumstances. In 2011, Wu and Zhou et al. presented an authentication protocol [10] which applied elliptic curve cryptography (ECC) into a smart grid to provide fault tolerance and scalability. However, it was soon pointed out that the scheme could not resist the man-in-the-middle attack. In 2012, Xia and Wang et al. proposed a new key distribution protocol [11] combined with the lightweight directory access protocol (LDAP), which improved both the security and the efficiency. Unfortunately, their scheme cannot resist impersonation attack and cannot guarantee the anonymity of users [12]. Also in 2012, Wang et al. presented an authentication protocol in identity [13], which ignores that smart meters need to be anonymous. Afterwards, Tsai and Lo [14] proposed a new smart grid authentication protocol using identity based encryption technology. However, their scheme was pointed out to be unable to guarantee the privacy of smart meter credentials [15], and cannot provide session key security under the widely accepted Canetti–Krawczyk adversary (CK-adversary) model [16,17]. In 2014, Nicanfar et al. suggested a security protocol [18] for authentication, with a key generator to update the grid’s key. However, his scheme’s computation cost is expensive, thus is not suitable for smart grids.

With the development of sensor equipment, lightweight authentication and key agreement schemes in smart grids have become popular. In 2018, Mohammadali et al. presented an identity-based authentication with light burden and key agreement scheme, where elliptic curve cryptography is used to establish a session key [19]. In the same year, Mahmood et al. also paid attention to the efficiency of the authentication scheme in a smart grid and designed a lightweight user authentication protocol [20] based on ECC. Unfortunately, although these two schemes are designed cleverly, they are not anonymous enough. Therefore, Kumar et al. [21] proposed a lightweight security protocol for anonymity in smart grids, but a synchronization problem was also pointed out. In 2019, Zhang et al. proposed an identity authentication communication scheme [22] in smart grids, which uses a novel dynamic verification table to protect the anonymous data, either in authentication or in the smart meters. Meanwhile, this new protocol is a fault-tolerant mechanism, and also ensures the untraceability of the user’s identity. In 2020, Ferrag et al. [23] pointed out that Zhang et al.’s scheme cannot ensure authentication of all nodes, but they did not give a detailed explanation. Sadhukhan et al. [24] showed the potential performance problems of Zhang et al.’s scheme. However, there are few articles on the security analysis of Zhang et al.’s scheme. In the same year, Abbasinezhad-Mood et al. proposed an anonymous ECC-based self-certified key distribution scheme [25], the scheme is not only free from the overhead of the certificate management and the key escrow issue, but is also more efficient than anonymous schemes in terms of both communication and computational costs.

In 2020, Khan et al. [26] suggested a novel system for smart grids PALK as a means for authenticating communication between two SG entities. Additionally, they analyzed many of PALK’s security characteristics and attack resilience. However, in 2022, Mohammed Taqi et al. [27] revealed that Khan et al.’s [26] scheme is vulnerable to well-known security threats, such as user anonymity and password guessing attack. They also proposed a new protocol LSPA-SG that they claimed would withstand every known attack. In 2022, Deebak et al. proposed a seamless authentication framework with privacy-preserving (SAF-PP) protocol [28] to deal with the security and privacy issues of smart eHealth intelligence. The formal analysis proves that SAF-PP can adhere to significant security properties while improving the system efficiency rate.

In a smart grid, where communication information is vulnerable to various attack threats, a secure authentication and key agreement protocol is essential for secure communication. We present a summary of the security problems and limitations of the recently available AKE schemes in Table 1. Meanwhile, most of the existing protocols [29,30,31] do not take into account the presence of insider attackers. In conjunction with the actual deployment environment of the smart grid, it is essential to consider how the protocol can guarantee the security of communication in the presence of insider attackers. In addition, it is necessary to consider a secure authentication protocol for IoT smart devices in resource-constrained scenarios in the smart grid.

### 1.2. Motivation

In the smart grid environment, it is critical to realize anonymity and untraceability for the smart meter with the presence of internal attackers during the authentication process. Zhang et al. [22] presented a practical authentication and key agreement scheme, and claimed that their proposal can satisfy security requirements and outperform the current solutions. This paper analyzes the security of Zhang et al.’s protocol [22], and points out that their scheme cannot really guarantee the claimed security requirements under their adversary model. More precisely, they regard the attacker as a third party, but ignore that the attacker may also be a legitimate consumer of the smart grid, who can obtain a legal identity of a smart meter.

Based on above assumption, we review and analyze Zhang et al.’s protocol [22] and point out that their work is not secure enough to prevent an impersonation attack from an insider attacker. We then improved Zhang et al.’s protocol so that it can resist against various attacks from both outsider and insider attackers.

### 1.3. Contribution

The contribution of this paper is as follows:

- We introduce an insider attack and take Zhang et al.’s protocol [22] as an example to improve the security. We first analyze the security of Zhang et al.’s protocol [22] and point out the reasons why their protocol cannot resist insider attack. Then, we describe the detailed steps of an insider attack, and show the potential threats of insider attack. In addition, we have made improvements to Zhang et al.’s protocol to resist insider attack.
- We analyze the security and performance of our proposed protocol and the results show that the protocol has strong security and efficiency. We analyze the security of the improved protocol in detail and prove the security of the protocol under the real-or-random oracle model. Through informal analysis, it has been shown that this protocol can resist common attacks, including insider attack. After that, we compare the proposed protocol with other schemes, indicating that the improved protocol is still lightweight.

The rest of the paper is introduced as follows. Section 3 analyzes the security of Zhang et al.’s scheme. In Section 3, an improved lightweight authentication and key agreement protocol is presented in details. Section 4 introduces the secure analysis of the improved scheme. In Section 5, a detailed comparison of the security and computational cost is conducted between the improved protocol and several other schemes. Section 7 concludes the whole paper.

## 2. Preliminaries

### 2.1. Communication Model

This paper focuses on the authentication between Service Provider and Customer, and thus it is based on the sub-network, which consists of the service provider and the smart meters. A smart meter is a hardware device deployed on the legitimate user side of the power grid, which can be used to transmit information in the power grid, undertake power consumption monitoring, electricity price information and other sensing functions [32,33,34]. Service providers provide power services for consumers, i.e., receive power information through SMs, charge the users, analyze the consumer’s data, and so on [35,36,37].

**Figure 2:**
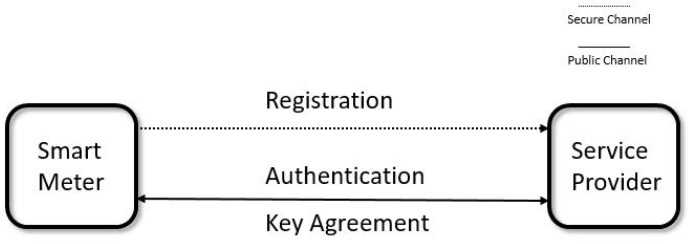
Communication model in smart grid.

A secure protocol is used between SMs and SPs to provide identity authentication and key agreement. A smart meter usually registers a legal identity through a secret channel before communication. After that, the SM and SP authenticate each other through the public network and generate a secret session key at the same time. The session key is used for SM and SP subsequent session encryption, as shown in Figure 2. From a practical point of view, a smart meter is easy to be access by anyone, and is thus vulnerable to eavesdropping, modification or physical attacks. Note that it is also a tamper resistant device so that the information stored in it is difficult to be stolen, changed or destroyed.

### 2.2. Threat Model

Assume an attacker is a probability polynomial time (PPT) attacker, which means he can obtain all the messages transmitted in the public channel, i.e., can intercept, modify or replay all the messages, can obtain access to all the normal released information in the grid, and can access and control smart meters, but cannot obtain sensitive data stored in the tamper-resistant devices. Moreover, according to Zhang et al. [22], an attacker can acquire SPs’ master keys or IDs, but not both. An attacker can also obtain the validation table used in the scheme from the providers’ servers.

### 2.3. Security Goals

The security scheme in smart grid should meet the following security objectives:

- Mutual authentication: Each session between the smart meter and service provider requires complete mutual authentication. This is to ensure that both sides of the communication are credible.
- Generate security session key: The security scheme should generate a temporary session key, which is confidential and unpredictable to any third party.
- Resist known security attacks: The security scheme should resist common attacks, such as man in the middle attack, replay attack, and so on.
- Provide SM’s anonymity: The scheme usually only needs to ensure that the third party cannot obtain the identity information of the smart meter. However, a good security protocol should make the service provider unable to figure out whether two sessions are possessed by the same SM or not.

## 3. Some Flaws of Zhang et al.’s Scheme

This section firstly exhibits Zhang et al.’s scheme, then shows how to mount an insider attack on Zhang et al.’s scheme.

### 3.1. Review of Zhang et al.’s Scheme

Zhang et al.’s scheme involves a registration phase and key agreement phase with authentication, which are executed by the smart meter $SM_{i}$ and the service provider $SP_{j}$.

#### 3.1.1. Registration Phase

- The smart meter $SM_{i}$ firstly chooses a random value with high entropy $r_{1}$. Then, $SM_{i}$ sends its identification $ID_{i}$ and $r_{1}$ to the service provider $SP_{j}$ securely.
- After receiving the messages from $SM_{i}$, $SP_{j}$ computes $Q_{i}=h(s\;⊕\;r_{1}\;⊕\;(ID_{j}||ID_{i}))$, where $ID_{j}$ means the identification of $SP_{j}$ and *s* means the master key of $SP_{j}$. Then, $SP_{j}$ calculates $M_{i}=E_{s}(\left(ID_{i}\;⊕\;r_{1}\right)||(h(ID_{j}\left||s\right)\;\;⊕\;\;ID_{i}))$ where $E_{s}\left(\right)$ means the secure symmetric encryption with secret key *s*. Finally, $SP_{j}$ stores $Q_{i}$ into its dynamic verification table and returns $M_{i}$ to $SM_{i}$ via a secure channel.
- $SM_{i}$ receives $M_{i}$ from $SP_{j}$, and stores $\left\{ID_{i},r_{1},M_{i}\right\}$ into its own tamper-resistant device.

#### 3.1.2. Authentication and Key Agreement Phase

- When the smart meter $SM_{i}$ attempts to start the communication with the service provider $SP_{j}$, $SM_{i}$ produces a random value with high entropy $r_{2}$ temporarily. After that, $SM_{i}$ calculates $X_{i}=h(ID_{i}\left|\right|r_{1})\;⊕\;r_{2}$ and sends $\left\{X_{i},M_{i}\right\}$ to $SP_{j}$ through a public channel.
- $SP_{j}$ decrypts $M_{i}$ with its master key *s* where $D_{s}\left(M_{i}\right)=\left({ID_{i}}^{\prime }\;⊕\;{r_{1}}^{\prime }\right)||(h({ID_{j}}^{\prime }\left|\right|s^{\prime })\;⊕\;{ID_{i}}^{\prime })$. Then, $SP_{j}$ calculates $ID_{i}^{*}=h(ID_{j}||s)\;⊕\;h({ID_{j}}^{\prime }\left|\right|s^{\prime })\;⊕\;{ID_{i}}^{\prime }$, $r_{1}^{*}=ID_{i}^{*}\;\;⊕\;\;{ID_{i}}^{\prime }\;⊕\;{r_{1}}^{\prime }$ and $Q_{i}^{*}=h(r_{1}^{*}\;⊕\;s\;⊕\;(ID_{j}||ID_{i}^{*}))$, and checks whether $Q_{i}^{*}$ is in its dynamic verification table or not. There are two columns, $Q_{i}$ and $Q_{i}o$, in the table, and the values of both columns are blank at the beginning. When $SM_{i}$ is successfully registered, the generated $Q_{i}$ would be stored in column $Q_{i}$. If $Q_{i}^{*}$ is found in $Q_{i}o$, $SP_{j}$ fills in the value of $Q_{i}o$ to $Q_{i}$. Once the value of $Q_{i}^{*}$ can be found in the table, $SP_{j}$ obtains $N_{2}^{*}=h(ID_{i}^{*}\left|\right|r_{1}^{*})\;⊕\;X_{i}$ and computes $M_{i}^{*}=E_{s}(\left(ID_{i}^{*}\;⊕\;r_{2}^{*}\right)||(h(ID_{j}\left||s\right)\;\;⊕\;\;ID_{i}^{*}))$. Next, $SP_{j}$ chooses a random value with high entropy $r_{3}$ and generates a temporary key $k=h(r_{1}^{*}\;\;⊕\;\;r_{2}^{*}\;⊕\;ID_{i}^{*})$. After this, $SP_{j}$ gets the session key $SK=h(r_{1}^{*}\;\;⊕\;\;r_{2}^{*}\;\;⊕\;\;r_{3}\;⊕\;ID_{i}^{*})$. To authenticate $SM_{i}$, $SP_{j}$ calculates $M_{2}=E_{k}(M_{i}^{*}||h(ID_{i}^{*}\left|\right|r_{1}^{*}\left|\right|r_{2}^{*})||(h(\left(ID_{i}^{*}\;⊕\;r_{2}^{*}\right)\left|\right|r_{1}^{*})\;⊕\;r_{3}))$ with the secret key *k* and returns $\left\{r_{2}\right\}$ to $SM_{i}$.
- $SM_{i}$ calculates $k^{*}=h\left(r_{1}\;⊕\;r_{2}\;⊕\;ID_{i}\right)$ and uses $k^{*}$ to decrypt $\left\{M_{2}\right\}$ that $D_{k^{*}}\left(M_{2}\right)=M_{i}^{**}||h(ID_{i}^{**}\left|\right|r_{1}^{**}\left|\right|r_{2}^{**})||(h(\left(ID_{i}^{**}\;⊕\;r_{2}^{**}\right)\left|\right|r_{1}^{**})\;⊕\;r_{3}^{*})$. Then, $SM_{i}$ checks whether $h(ID_{i}^{**}\left|\right|r_{1}^{**}\left|\right|r_{2}^{**})=h(ID_{i}\left|\right|r_{1}\left|\right|r_{2})$ or not. If this verification is valid, $SM_{i}$ computes $r_{3}^{*}=(h(\left(ID_{i}\;⊕\;r_{2}\right)\left|\right|r_{1})\;⊕\;(h(\left(ID_{i}^{**}\;⊕\;r_{2}^{**}\right)\left|\right|r_{1}^{**})\;⊕\;r_{3}^{*}$. Next, $SM_{i}$ computes $SK^{*}=h\left(r_{1}\;⊕\;r_{2}\;⊕\;r_{3}^{*}\;⊕\;ID_{i}\right)$ and sends $M_{3}=h(SK^{*}\left|\right|r_{3}^{*})$ to $SP_{j}$.
- After receiving $M_{3}$ from $SM_{i}$, $SP_{j}$ checks whether $h(SK||r_{3})$ matches with $M_{3}$. If it holds, $SP_{j}$ displaces $\left(Q_{i},Q_{i}o\right)$ with $\left(Q_{i}new,Q_{i}\right)$ where $Q_{i}new=h(s\;⊕\;r_{2}\;⊕\;(ID_{j}||ID_{i}^{*}))$. Then, $SP_{j}$ calculates $M_{4}=h(r_{1}^{*}\left|\right|\left(r_{2}^{*}\;⊕\;r_{3}\right))$ and returns $\left\{M_{4}\right\}$ to $SM_{i}$.
- $SM_{i}$ checks whether $h(r_{1}||\left(r_{2}\;⊕\;r_{3}^{*}\right)=M_{4})$. If this verification is valid, $SM_{i}$ replaces $\left(r_{1},M_{i}\right)$ with $\left(r_{2},M_{i}^{**}\right)$. After that, $SM_{i}$ and $SP_{j}$ apply the session key $SK=SK^{*}$ into the communication.

### 3.2. Security Flaws of Zhang et al.’s Scheme

Under the assumption of the threat model in Zhang et al.’s scheme [22], an adversary is able to obtain either the service providers’ master key or ID, but not both. Unfortunately, in the smart grid, the attacker is likely to be a insider attack, which is introduced by Kumar et al. [4] in 2019. An insider attacker A can not only intercept messages through the channel, but also register as a legitimate user.

Next, we look into Zhang et al.’s scheme and show how an insider attacker attacks the scheme step by step using the identity of consumers and the master key *s*.

**Figure 3:**
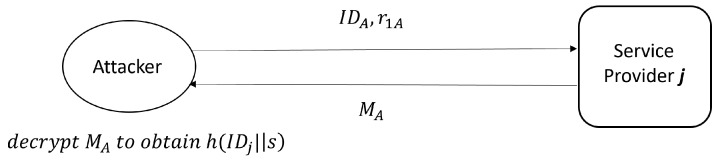
Attacker registration as a normal user.

**Figure 4:**
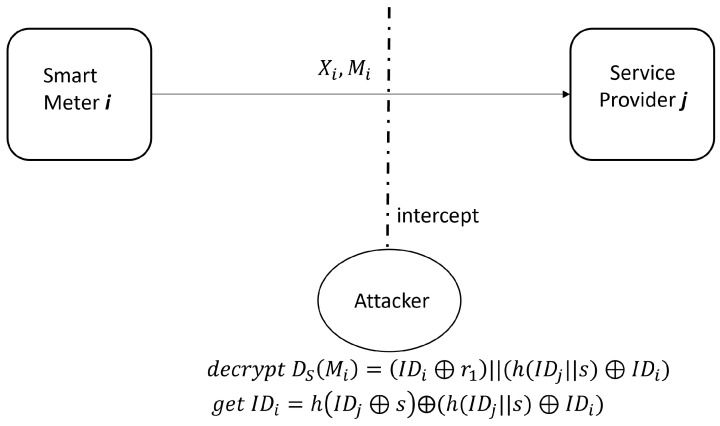
Attacker intercepts on $SM_{i}$’s information.

- Step 1. The attacker firstly registers to $SP_{j}$ as a legitimate user, and obtains his identity information $ID_{A}$, $r_{1A}$ and $M_{A}$ after successful registration. He/she can decrypt $M_{A}$ with key *s* to obtain $(h({ID_{j}}^{\prime }\left|\right|s^{\prime })\;⊕\;ID_{A})$, where $ID_{A}={ID_{A}}^{\prime }$. The phase is shown in Figure 3.
- Step 2. During the protocol process between a registered smart meter $SM_{i}$ and $SP_{j}$, the attacker intercepts $\left\{X_{i},M_{i}\right\}$ sent by $SM_{i}$ to $SP_{j}$. Then, he/she decrypts the information $M_{i}$ with key *s* to obtain $\left({ID_{i}}^{\prime }\;⊕\;{r_{1}}^{\prime }\right)$ and $(h({ID_{j}}^{\prime }\left|\right|s^{\prime })\;⊕\;{ID_{i}}^{\prime })$, where $ID_{i}={ID_{i}}^{\prime }$ generally.
- Step 3. The attacker computes $ID_{i}=(h({ID_{j}}^{\prime }\left|\right|s^{\prime })\;⊕\;ID_{i})\;⊕\;(h({ID_{j}}^{\prime }\left|\right|s^{\prime })\;⊕\;ID_{A})\;⊕\;ID_{A}$, where $(h({ID_{j}}^{\prime }\left|\right|s^{\prime })\;⊕\;ID_{i})$ comes from step 2 and $(h({ID_{j}}^{\prime }\left|\right|s^{\prime })\;⊕\;ID_{A})$ comes from step 1. This phase is shown in Figure 4.

At this time, the attacker has successfully attacked the legitimate $SM_{i}$. The attacker may then use, but is not limited to, the following means of attacks.

- The loss of smart meter anonymity: Through Step 3 above, the attacker obtains $ID_{i}$ of legitimate $SM_{i}$, which directly leads to the loss of the user’s anonymity.
- Impersonate attack: The attacker can also obtain $r_{1}$ stored in $SM_{i}$ by calculating $r_{1}=\left({ID_{i}}^{\prime }\;⊕\;{r_{1}}^{\prime }\right)\;⊕\;ID_{i}$ and $M_{i}$ from the intercepted message. This means that all information $\left\{ID_{i},r_{1},M_{i}\right\}$ stored by $SM_{i}$ is obtained by the attacker. The attacker can impersonate $SM_{i}$ and communicate with $SP_{j}$.
- Session key compromise attack: If the attacker knows the information $\left\{ID_{i},r_{1},M_{i}\right\}$, he/she can compute $X_{i}=h(ID_{i}\left|\right|r_{1})\;⊕\;r_{2}$ and fake $SM_{i}$ by sending $\left\{X_{i},M_{i}\right\}$ to $SP_{j}$. After receiving the message $\left\{M_{2}\right\}$ from $SP_{j}$, the attacker is able to execute all operations performed by $SM_{i}$ step by step, and gets the correct $SK^{*}$ and returns correct $M_{3}$ to $SP_{j}$.
- Permanent denial of service attack: Because the scheme uses a dynamic authentication table to verify $SM_{i}$’s identity, $SM_{i}$ will lose the ability of communication with $SP_{j}$ permanently when the attacker disguises $SM_{i}$ twice in succession. In abstract, this scheme only retains the current and last session credentials of $SM_{i}$ in the verification table of $SP_{j}$. Once the attacker successfully disguises $SM_{i}$ more than two times, the session credentials of $SM_{i}$ will expire, and this process is irreversible.

### 3.3. Reasons for the Weakness

The direct reason for the weakness of Zhang et al.’s scheme [22] is that $SP_{j}$ assigns the same information for all smart meters, which is used to meet the untraceability and the anonymity of smart meters. However, this also results in the consequence that $SP_{j}$ is unable to store the identity credentials used to distinguish different smart meters. To authenticate the smart meter $SM_{i}$ successfully, $SP_{j}$ has to know *s* and $h(ID_{j}||s)$ for obtaining $ID_{i}$. Unfortunately, the insider attacker can also take advantage of this to obtain $SM_{i}$’s identity information, i.e., an attacker A obtains the certificate $h(ID_{j}||s)$ at the time of his registration through his legal identity, and then uses $h(ID_{j}||s)$ to obtain the authentication information of other SMs, and finally realizes the insider attack.

## 4. The Improved Scheme

To avoid the above insider attack, different SMs should have different authentication information. Note that the anonymity of the SM cannot be destroyed when distinguishing different SMs. Based on this analysis, this section presents an improved protocol, which involves two phases: a registration phase, and an authentication and key agreement phase. Table 2 lists the notations used in the improved scheme.

### 4.1. Registration Phase

**Figure 5:**
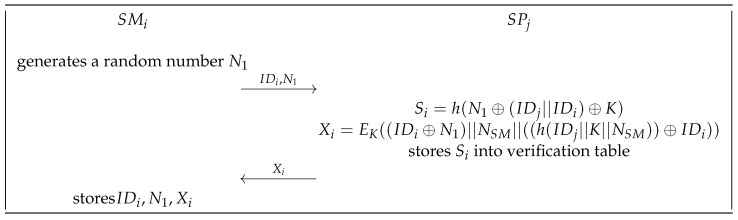
Registration phase.

Before $SM_{i}$ communicates with $SP_{j}$, $SM_{i}$ firstly applies to $SP_{j}$ for registration, as shown in Figure 5.

**Figure 6:**
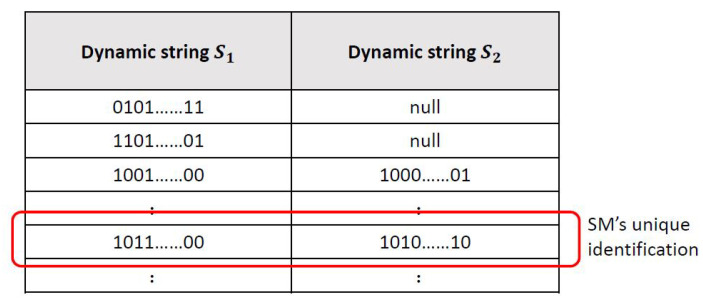
The dynamic verification table.

- The smart meter $SM_{i}$ firstly generates a random integer $N_{1}$. Then, $SM_{i}$ sends its identification $ID_{i}$ and $N_{1}$ to $SP_{j}$ via a secure channel.
- After receiving the message from $SM_{i}$, $SP_{j}$ computes $S_{i}=h(N_{1}\;⊕\;(ID_{j}||ID_{i})\;⊕\;K)$, where *K* means the master key of the service providers. Next, $SP_{j}$ calculates $X_{i}=E_{K}(\left(ID_{i}\;⊕\;N_{1}\right)\left|\right|N_{SM}||((h(ID_{j}\left||K|\right|N_{SM}))\;⊕\;ID_{i}))$, where $N_{SM}$ is a high entropy random number generated for $SM_{i}$. Then, $SP_{j}$ stores $S_{i}$ into its dynamic verification table. The dynamic verification table is shown in Figure 6. There are two columns $S_{1}$ and $S_{2}$ in the table, and the values of both columns are blank at the beginning. When $SM_{i}$ is successfully registered, the generated $S_{i}$ will be stored in a new blank of column $S_{1}$. Each row in the table represents a registered smart meter. Finally, $SP_{j}$ returns $X_{i}$ to $SM_{i}$ securely.
- $SM_{i}$ receives $X_{i}$ from $SP_{j}$, and stores $\left\{ID_{i},N_{1},X_{i}\right\}$ into its own tamper-resistant device.

### 4.2. Authentication and Key Agreement Phase

**Figure 7:**
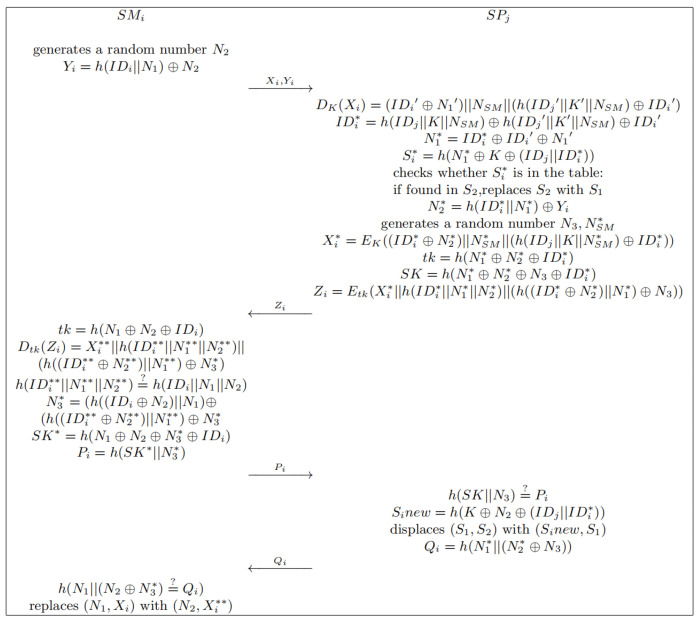
Authentication and key agreement phase.

When $SM_{i}$ attempts to set up a new session with $SP_{j}$, it needs to perform the following steps. The details of the whole session are shown in Figure 7.

- When $SM_{i}$ is willing to build the communication with the $SP_{j}$, $SM_{i}$ needs to produce a high entropy random number $N_{2}$ temporarily. After that, $SM_{i}$ calculates $Y_{i}=h(ID_{i}\left|\right|N_{1})\;⊕\;N_{2}$ and sends $\left\{X_{i},Y_{i}\right\}$ to $SP_{j}$ through a public channel.
- $SP_{j}$ decrypts $M_{i}$ with its master key *K* where $D_{K}\left(X_{i}\right)=\left({ID_{i}}^{\prime }\;⊕\;{N_{1}}^{\prime }\right)\left|\right|N_{SM}||(h({ID_{j}}^{\prime }\left|\right|K^{\prime }$$\left|\right|N_{SM})\;⊕\;{ID_{i}}^{\prime })$. Then, $SP_{j}$ can calculate $ID_{i}^{*}=h(ID_{j}\left||K|\right|N_{SM})\;⊕\;h({ID_{j}}^{\prime }\left|\right|K^{\prime }\left|\right|N_{SM})\;⊕\;{ID_{i}}^{\prime }$, $N_{1}^{*}=ID_{i}^{*}\;⊕\;{ID_{i}}^{\prime }\;⊕\;{N_{1}}^{\prime }$ and $S_{i}^{*}=h(N_{1}^{*}\;⊕\;K\;⊕\;(ID_{j}||ID_{i}^{*}))$, and checks whether $S_{i}^{*}$ can be found in its dynamic verification table or not. If $S_{i}^{*}$ is found in $S_{2}$, $SP_{j}$ fills in the value of $S_{2}$ to $S_{1}$. Once the value of $S_{i}^{*}$ can be found in the table, $SP_{j}$ can obtain $N_{2}^{*}=h(ID_{i}^{*}\left|\right|N_{1}^{*})\;⊕\;Y_{i}$. Next, $SP_{j}$ chooses high entropy random numbers $N_{3}$ and $N_{SM}^{*}$. It can compute $X_{i}^{*}=E_{K}(\left(ID_{i}^{*}\;⊕\;N_{2}^{*}\right)\left|\right|N_{SM}^{*}||(h(ID_{j}\left||K|\right|N_{SM}^{*})\;⊕\;ID_{i}^{*}))$ and generate a temporary key $tk=h(N_{1}^{*}\;⊕\;N_{2}^{*}\;⊕\;ID_{i}^{*})$. After this, $SP_{j}$ can obtain the session key $SK=h(N_{1}^{*}\;⊕\;N_{2}^{*}\;⊕\;N_{3}\;⊕\;ID_{i}^{*})$. To authenticate $SM_{i}$, $SP_{j}$ calculates $Z_{i}=E_{tk}(X_{i}^{*}||h(ID_{i}^{*}\left|\right|N_{1}^{*}\left|\right|N_{2}^{*})||(h(\left(ID_{i}^{*}\;⊕\;N_{2}^{*}\right)\left|\right|N_{1}^{*})\;⊕\;N_{3}))$ with secret key tk and returns $\left\{Z_{i}\right\}$ to $SM_{i}$.
- $SM_{i}$ calculates $tk=h(N_{1}\;⊕\;N_{2}\;⊕\;ID_{i})$ and uses tk to decrypt $\left\{Z_{i}\right\}$ that $D_{tk}\left(Z_{i}\right)=X_{i}^{**}||h(ID_{i}^{**}\left|\right|N_{1}^{**}\left|\right|N_{2}^{**})||(h(\left(ID_{i}^{**}\;⊕\;N_{2}^{**}\right)\left|\right|N_{1}^{**})\;⊕\;N_{3}^{*})$. Then, $SM_{i}$ checks whether $h(ID_{i}^{**}\left|\right|N_{1}^{**}\left|\right|N_{2}^{**})=h(ID_{i}\left|\right|N_{1}\left|\right|N_{2})$ or not. If this verification is valid, $SM_{i}$ can compute $N_{3}^{*}=(h(\left(ID_{i}\;⊕\;N_{2}\right)\left|\right|N_{1})\;⊕\;(h(\left(ID_{i}^{**}\;⊕\;N_{2}^{**}\right)\left|\right|N_{1}^{**})\;⊕\;N_{3}^{*}$. Next, $SM_{i}$ computes $SK^{*}=h\left(N_{1}\;⊕\;N_{2}\;⊕\;N_{3}^{*}\;⊕\;ID_{i}\right)$ and sends $P_{i}=h(SK^{*}\left|\right|N_{3}^{*})$ to $SP_{j}$.
- After receiving message $P_{i}$ from $SM_{i}$, $SP_{j}$ makes a check on whether $h(SK||N_{3})$ matches with $P_{i}$. If it holds, $SP_{j}$ displaces $\left(S_{1},S_{2}\right)$ with $\left(S_{i}new,S_{1}\right)$ where $S_{i}new=h(K\;⊕\;N_{2}\;⊕\;(ID_{j}||ID_{i}^{*}))$. Then, $SP_{j}$ calculates $Q_{i}=h(N_{1}^{*}\left|\right|\left(N_{2}^{*}\;⊕\;N_{3}\right))$ and returns $\left\{Q_{i}\right\}$ to $SM_{i}$.
- $SM_{i}$ checks whether $h(N_{1}||\left(N_{2}\;⊕\;N_{3}^{*}\right)=Q_{i})$. If this verification is valid, $SM_{i}$ replaces $\left(N_{1},X_{i}\right)$ with $\left(N_{2},X_{i}^{**}\right)$. After that, $SM_{i}$ and $SP_{j}$ apply the session key $SK=SK^{*}$ in the communication.

## 5. Security Analysis of the Improved Scheme

### 5.1. Formal Security Analysis

This section presents the rigorous security analysis of the improved scheme in formal. First, the security model of the protocol is proposed.

#### Security Model

Suppose the attacker can control the messages transmitted on all public channels and obtain all public parameters. An attacker cannot obtain secret information (for example, information in the tamper-resistant device), but can obtain some leaked information (for example, either the SP’s master key or the SP’s identity). There are two kinds of identity, i.e., the smart meters’ identities and the service providers’ identities, where $SM_{i}$ is for a smart meter’s identity and $SP_{j}$ is for a service provider’s identity. The improved protocol’s security is proved in the real-or-random oracle model, and the details are represented by *O*. *E* is used to represent $SM_{i}$ or $SP_{j}$.

- Execute ($SM_{i}$, $SP_{j}$): This module is built to simulate a passive attack. An attacker can obtain all messages sent by $SM_{i}$ and $SP_{j}$ through this operation.
- Send (*E*, *M*): This operation aims at simulating an active attack. An attacker can send message *M* to any $SM_{i}$ or $SP_{j}$, and $SM_{i}$ or $SP_{j}$ would perform corresponding steps according to the protocol. The corresponding information would also be returned to the attacker.
- Corrupt (*E*): This operation is applied to simulate the forward security. The attacker can obtain the leaked long-term secret information, such as $SP_{j}$’s master key or $SP_{j}$’s identity (but not both).
- Test (*E*): This operation is used to return the session key or a randomly generated key. This response depends on a random bit *b*. If *b* is equal to 1, the query returns the session key; if *b* equals 0, the query returns a random key.

Semantic security: An attacker A can perform several test (*E*) operations. Each time, a key is obtained based on the result of *b*. This process indicates that the attacker distinguishes between the session key and the random key. Pr[Success] is the probability of the attacker to be the game winner, which is expressed as follows:

$$Adv_{A}^{ake}=\left|2Pr\left[Succ\right]-1\right|$$

Now we will prove that in the improved protocol, the attacker’s advantage is non-negligible. We first show the difference lemma [38].

**Lemma 1**  (Difference Lemma). *Let X, Y and Z represent the events defined in some probability distribution. If X∧¬Z⇔Y∧¬Z, |Pr[X]−Pr[Y]|≤Pr[Z].*

**Theorem 1.**  *Let $Adv_{A}^{se}$ represent the advantage of A in breaking the symmetric cipher algorithm, and l represent a security parameter. Let $q_{send}$ and $q_{c}$ represent the upper limit of hash queries when simulating an active attack and guessing key tk, respectively. The advantage of A breaking the semantic security is*

$$Adv_{A}^{ake}\leq 2Adv_{A}^{se}+\left(q_{c}^{2}+3q_{send}^{2}\right)/2^{l}$$

**Proof.**  We define the sequence of games $GM_{0}$ to $GM_{3}$. Let $Succ_{i}$ be the event that A guesses bit *b* for $GM_{i}$ in the test session successfully. The games $GM_{0}$ to $GM_{3}$ are presented as follows.

**Game $GM_{0}$:** This game is related to the real attack under the random oracle model. Therefore

$$Adv_{A}^{ake}=2\left|Pr\left[Succ_{0}\right]-1/2\right|$$

**Game $GM_{1}$:** We query Execute oracles several times to simulate A’s eavesdropping attack. Because all messages are encrypted symmetrically or hashed, these operations cannot make A obtain more useful information. Thus A cannot extend the advantage of winning game $GM_{1}$. Therefore

$$Pr\left[Succ_{0}\right]=Pr\left[Succ_{1}\right]$$

**Game $GM_{2}$:** We query Send and Hash oracles to simulate A’s active attack. It is impossible for A to find the collisions of $Y_{i}/P_{i}/Q_{i}$ in the way of making queries, or decrypting the information of $X_{i}/Z_{i}$ without key K/tk. Hence there is no collision when querying Send oracles. Due to the birthday paradox, we obtain

$$|Pr\left[Succ_{2}\right]-Pr\left[Succ_{1}\right]|\leq Adv_{A}^{se}+3q_{send}^{2}/2^{l+1}$$

**Game $GM_{3}$:** This game aims at simulating forward security using the Corrupt oracle query. Even if A is lucky enough to find the correct hash collision value, he still needs to find a way to obtain the long-term stored key *K* and the temporarily generated tk. Thus, A has to query the Corrupt and Hash Oracle, and we have

$$|Pr\left[Succ_{3}\right]-Pr\left[Succ_{2}\right]|\leq q_{c}^{2}/2^{l+1}$$

A cannot receive any useful messages since all the oracles have been simulated. At this point, A has half the chance to guess the correct value of *b*, so the probability to win $GM_{3}$ is:

$$Pr\left[Succ_{3}\right]=\frac{1}{2}$$

As a result, the final output performs as follows:

$$Adv_{A}^{ake}\leq 2Adv_{A}^{se}+\left(q_{c}^{2}+3q_{send}^{2}\right)/2^{l}$$

The semantic security of the improved protocol is proved completely. □

### 5.2. Informal Security Analysis

This subsection contains an informal security analysis to show that the improved protocol can resist various attacks from both outsider and insider attackers.

#### 5.2.1. Insider Attack

As mentioned in Section 2, an attacker A may have a legitimate identity. Even if A obtains the master key *K* of the service provider, A cannot pretend to be another consumer in the improved protocol, since $N_{SM}$ is stored in $X_{i}=E_{K}(\left(ID_{i}\;⊕\;N_{1}\right)\left|\right|N_{SM}||((h(ID_{j}\left||K|\right|N_{SM}))$$\;⊕\;ID_{i}))$ during the registration phase, and $(h(ID_{j}\left||K|\right|N_{SM})$ generated by each consumer is different. Although $N_{SM}$ can be obtained when decrypting $X_{i}$ with key *K*, $(h(ID_{j}\left||K|\right|N_{SM})$ cannot be calculated without $ID_{j}$ or *K* at the same time. In addition, $SP_{j}$ still does not store $SM_{i}$’s ID so it still meets $SM_{i}$’s non-traceability.

#### 5.2.2. De-Synchronization Attack

Our scheme can resist de-synchronization attacks with a dynamic verification table. If A intercepts message $X_{i},Y_{i}$, $Z_{i}$ or $P_{i}$, $SM_{i}$’s unique identification in the table is not updated, and $SM_{i}$ can perform the authentication phase again. Suppose that A intercepts message $Q_{i}$ sent by $SP_{j}$ and $S_{1}$ of the dynamic verification table has been updated, while the last unique identification is also stored in $S_{2}$. Once $SM_{i}$ has not received the confirmation message within the limited time, it can still use the last unique identification to authenticate, which can be found in $S_{2}$.

#### 5.2.3. Replay Attack

If A wants to replay messages $\left\{X_{i},Y_{i}\right\}$ sent by $SM_{i}$, it can pass the first step of $SP_{j}$’s verification. However, after receiving message $Z_{i}$ returned by $SP_{j}$, A cannot generate $tk=h(N_{1}^{*}\;⊕\;N_{2}^{*}\;⊕\;ID_{i}^{*})$. The attacker will not succeed in replaying message $P_{i}$, because $N_{3}$ generated by $SP_{j}$ is different each time. If A wants to replay the messages $Z_{i}$ and $Q_{i}$ sent by $SP_{j}$, $SM_{i}$ will find that $h(ID_{i}||N_{1}||N_{2})$ and $h(N_{1}||\left(N_{2}\;⊕\;N_{3}^{*}\right))$ are not the same, since $N_{2}$ is randomly generated by $SM_{i}$ for each session.

#### 5.2.4. Man-in-the-Middle Attack

The messages sent by $SM_{i}$ and $SP_{j}$ are processed by hash function or symmetrically encrypted. Even if A obtains the master key *K*, he can only decrypt the first message $X_{i}$ sent by $SM_{i}$ and cannot obtain other useful information. Because of the anonymity of $SM_{i}$, A cannot obtain $ID_{i}$ of $SM_{i}$. The random number $N_{1}$ is also not be obtained because the tamper-resistant device prevents its leakage. Therefore, even if the attacker modifies the message, A cannot mount a Man-in-the-middle attack.

#### 5.2.5. Impersonation Attack

In the impersonation attack, we consider an adversary A, who can monitor the public network and capture the message $\left\{X_{i},Y_{i},Z_{i},P_{i},Q_{i}\right\}$ transferred on an insecure channel. We consider two cases.

- SM impersonation attack. If adversary A wants to impersonate the SM, he must forge the message $\left\{X_{i},Y_{i},P_{i}\right\}$ to have the SP believe that the message is legal. However, A must know secret parameters such as $ID_{i},N_{1},N_{3}$ in order to produce the messages as legal. Due to the lack of knowledge of these parameters, the adversary cannot implement the SM impersonation attack.
- SP impersonation attack. If the adversary A wants to impersonate SP, it needs to forge the message $\left\{Z_{i},Q_{i}\right\}$. The generation of the message requires SP’s long-term secret as auxiliary material which the adversary cannot learn. Therefore, the improved scheme can resist the impersonation attack.

#### 5.2.6. Anonymity and Untraceability

The purpose of anonymity is to prevent the adversary, who can intercept messages in an insecure channel, from obtaining the actual ID of the smart grid. At a higher level, the adversary cannot find any relationship among sessions of one entity. In the improved scheme, all the transmitted messages are $\left\{X_{i},Y_{i},Z_{i},P_{i},Q_{i}\right\}$. The ID of SM is sent over the insecure channel with the use of a hash function. Therefore, the adversary cannot derive the actual ID from the transmitted messages. Furthermore, each communicated message is dynamically changed by involving random numbers. The adversary also fails to trace the participants.

#### 5.2.7. Perfect Forward Secrecy

This property means even if the long-term secret parameters of both entities are leaked, it will not lead to the previous session key being compromised. In our protocol, if the long-term secret parameters of SM and SP are compromised, the adversary needs to know the ephemeral secret to compute. Due to the lack of the knowledge of the ephemeral secret, the session key remain secure.

## 6. Performance Analysis

How to improve security without affecting the efficiency is one of the goals of improving the protocol. This section presents a comparison between the improved protocol and four other protocols proposed recently for smart grids [19,20,21,22,27], in terms of functionality, computation efficiency and the communication efficiency.

### 6.1. Security and Functionality Analysis

Table 3 shows the comparison of security and functionality, where F1 denotes mutual authentication, F2 denotes session-key security, F3 denotes message integrity, F4 denotes smart meters’ anonymity, F5 denotes perfect forward secrecy, F6 denotes smart meters’ untraceability, R1 denotes the ability to resist replay attack, R2 denotes resistance to man-in-the-middle attack, R3 denotes resistance to impersonation attack, R4 denotes resistance to de-synchronization attack, R5 denotes resistance to insider attack and R6 denotes resistance to stolen verifier attack.

From Table 3, it can be seen that the improved scheme can meet the basic functionality and security requirements. In terms of functionality, our scheme can guarantee mutual authentication, session-key security, message integrity, perfect forward secrecy, smart meters’ anonymity and untraceability. In terms of security, our scheme can resist replay attacks, man-in-the-middle attacks, impersonation attack, de-synchronization attacks, insider attacks and stolen verifier attacks. In addition, some protocols do not consider insider attackers [19,21,22]. Some schemes [19,20,22] do not provide smart meters’ anonymity, while some [19,20,21,27] cannot resist de-synchronization attacks. To summarize, our improved protocol has advantages in security and functionality.

### 6.2. Computation Overhead Analysis

Only the authentication and agreement phase is compared when analyzing the computation cost, since this phase is the main part of the scheme. The executions of the concatenating operation and OR operation are not considered, because the time of these executions is negligible. Let $T_{h}$ denote the computation time for the one-way hash function, $T_{e}$ denote the computation time for symmetric encryption operation, $T_{d}$ denote the computation time for a symmetric decryption operation, $T_{HMAC}$ denote the computation time for the hash-based message authentication code (HMAC) operation, $T_{a}$ denote the computation time for point addition of elliptic curve and $T_{m}$ denote the computation time for the point multiplication of the elliptic curve. According to the literature of Abbasinezhad-Mood et al. [39], we have that $T_{h}$ takes 0.0023 ms, $T_{e}$ takes 0.0046 ms, $T_{d}$ takes 0.0046 ms, $T_{HMAC}$ takes 0.0046 ms, $T_{a}$ takes 0.0288 ms and $T_{m}$ takes 2.226 ms. Let C1 denote the computation cost in the smart meter, C2 denote the computation overhead in the service provider phase and C3 denote the total costs. Table 4 shows the computational cost including the improved protocol and other ones.

It can be seen from Table 4 that the computation cost of the improved protocol at the smart meter is $C_{1}=7T_{h}+1T_{d}$, i.e., the smart meter needs to execute seven one-way hash function operations and an encryption operation. In addition, the service provider’s computation overhead is $C_{2}=9T_{h}+2T_{e}+1T_{d}$. The total cost is $C_{3}=16T_{h}+2T_{d}+2T_{e}$, which is less computation resources than the protocols using the elliptic curve [19,20,21,27]. The new protocol has the same computation efficiency as the original protocol [22]. Both of them are 0.0552 ms. Since these two protocols have a great improvement in computation cost compared with the known protocols [19,20,21,27], our proposed protocol is still lightweight in terms of computational overhead level.

### 6.3. Communication Overhead Analysis

This subsection discusses the communication cost of the improved protocol. Suppose the hash function used in the improved protocol is SHA1 and the symmetric encryption algorithm is AES-128. The output of the hash function is 20 bytes (160 bits), the output of a 128 bit AES is based on the input of the plaintext, and the random number is 128 bits long. Similarly, a point of the elliptic curve is assumed to be 40 bytes (320 bits).

Let C4 denote the communication cost in the authentication and key agreement phase. Table 5 shows the communication cost including the improved protocol and others.

Table 5 shows that the communication cost of these protocols are relatively close, ranging from 200 bytes to 300 bytes. The new protocol has a communication cost of 236 bytes, which is a little higher than the original protocol [22]. However, it is still less than the communication cost of protocols [19,20,21,27]. In other words, this communication cost is acceptable in smart grids.

To sum up, the improved protocol obtains the strongest security against both the outsider attacker and the insider attacker, and is the only one obtaining all security properties. In terms of the computation efficiency and the communication efficiency, the improved protocol has low computational cost and communication cost compared with the lightweight schemes, thus is very suitable for the smart grid with limited computing resources.

## 7. Conclusions

In this paper, we analyzed the security of Zhang et al.’s protocol [22] and showed that their protocol is not secure enough to prevent an impersonation attack against an insider attacker, since different SMs hold the same confidential information. To address the flaws, we proposed an improved protocol which allows SMs and SPs to authenticate each other and establish a session key securely. Moreover, we verified the security of the improved protocol using the ROR model. By conducting an informal security analysis, we demonstrated that the new protocol is secure against various attacks from both outsider and insider attackers. In terms of the computation cost and communication cost, the improved protocol has almost the same efficiency as Zhang et al.’s protocol, while providing enhanced security. Furthermore, the proposed protocol has a significantly lower computational cost compared to the other protocols, which is well suited to smart grid environments where smart meters are resources-constrained. The limitation of our protocol is that it is not suitable in general communication models, except wireless ones. In addition, our protocol has no significant advantage over existing schemes in terms of communication cost. We will consider these two limitations in our future work.

## Figures and Tables

**Table 1:** Overview of authentication protocols.

Scheme	Limitations	Primitive Used	Year
[19]	Cannot resist replay attack, SK compromised attack, SM impersonation attack, and MINM attacks, cannot ensure anonymity, does not consider internal attackers	ECC, XOR, and Hash	2018
[20]	Cannot ensure the anonymity of SM, does not consider internal attackers	ECC, XOR, and Hash	2018
[21]	Cannot ensure the anonymity of SM, does not consider internal attackers	ECC, XOR, and Hash	2018
[22]	Cannot resist insider attacks, impersonation attack, SK compromise attack, loss the anonymity of SM	AES, XOR, and Hash	2019
[27]	Does not consider internal attackers	ECC, XOR, and Hash	2022
our scheme	Can resist known attacks, considers internal attackers	AES, XOR, and Hash	

**Table 2:** Symbols and notations in our scheme.

Symbols and Notations	Description
$SM_{i},ID_{i}$	the *i*th smart meter and $SM_{i}$’s identity
$SP_{j},ID_{j}$	the *j*th service provider and $SP_{j}$’s identity
*K*	service providers’ master secret key
tk	a temporary key between $SM_{i}$ and $SP_{j}$
||	concatenating operation
⊕	exclusive OR
$h(\dot{)}$	a one-way hash function
$E_{K}$	Symmetric encryption using key K
$D_{K}$	Symmetric decryption using key K
$N_{1},N_{2},N_{3},N_{SM}$	High entropy random numbers
$S_{i}$	$SM_{i}$’s unique identification stored in the table

**Table 3:** Security and functionality comparison table.

	*F*1	*F*2	*F*3	*F*4	*F*5	*F*6	*R*1	*R*2	*R*3	*R*4	*R*5	*R*6
[19]	YES	YES	YES	NO	NO	YES	YES	YES	YES	/	/	YES
[20]	YES	YES	YES	NO	NO	YES	YES	YES	YES	/	YES	YES
[21]	YES	YES	YES	YES	/	YES	YES	YES	NO	/	/	YES
[22]	YES	YES	YES	NO	YES	YES	YES	YES	YES	YES	NO	YES
[27]	YES	YES	YES	YES	YES	YES	YES	YES	YES	NO	YES	YES
our scheme	YES	YES	YES	YES	YES	YES	YES	YES	YES	YES	YES	YES

**Table 4:** Computation cost comparison table.

	[19]	[20]	[21]	[22]	[27]	Our Scheme
C1	3$T_{h}\;$+ 2$T_{m}$ =	3$T_{h}$ + 2$T_{m}$ =	3$T_{h}$ + 2$T_{m}$ + $T_{e}$ + $T_{d}$ +	7$T_{h}$ + 1$T_{d}$ =	4$T_{h}$ + $T_{e}$ + $T_{d}$ + 3$T_{m}$	7$T_{h}$ + 1$T_{d}$ =
	4.4589 ms	4.4589 ms	$2T_{HMAC}$ = 4.4773 ms	0.0207 ms	= 6.7796 ms	0.0207 ms
C2	4$T_{h}$ + 3$T_{m}$ =	2$T_{h}$ + 3$T_{m}$ + $T_{a}$	4$T_{h}$ + 3$T_{m}$ + $T_{e}$ + $T_{d}$ +	9$T_{h}$ + 2$T_{e}$ + $T_{d}$	3$T_{h}$ + $T_{e}$ + $T_{d}$ + 3$T_{m}$	9$T_{h}$ + 2$T_{e}$ + $T_{d}$
	6.6872 ms	= 6.7114 ms	2$T_{HMAC}$ = 6.7056 ms	= 0.0345 ms	= 6.7772 ms	= 0.0345 ms
C3	11.1461 ms	11.1703 ms	11.1829 ms	0.0552 ms	13.5568 ms	0.0552 ms

**Table 5:** Communication cost comparison table.

	[19]	[20]	[21]	[22]	[27]	Our Scheme
C4	248 bytes	298 bytes	254 bytes	204 bytes	248 bytes	236 bytes

## Data Availability

Not applicable.
